# Dihydroartemisinin Promotes the Osteogenesis of Human Mesenchymal Stem Cells via the ERK and Wnt/*β*-Catenin Signaling Pathways

**DOI:** 10.1155/2019/3456719

**Published:** 2019-08-26

**Authors:** Licheng Ni, Zhihui Kuang, Zhe Gong, Deting Xue, Qiang Zheng

**Affiliations:** ^1^Department of Orthopedics, 2nd Affiliated Hospital, School of Medicine, Zhejiang University, #88 Jiefang Road, Hangzhou, 310009 Zhejiang, China; ^2^Orthopedics Research Institute of Zhejiang University, No. 88, Jiefang Road, Hangzhou 310009, China

## Abstract

Dihydroartemisinin (DHA), which is considered to be one of the active compounds within Artemisia annua, has extensively been used in recent years as the most effective drug against malaria, having many biological functions including anticancer, antifungal, and immunomodulatory activities. However, DHA plays a role in the regulation of the proliferation and human mesenchymal stem cells (hMSCs) osteogenic differentiation that remains unknown. We explored DHA's effect on hMSCs' proliferation as well as the osteogenic differentiation, together with its underlying mechanisms of action. We showed that DHA enhanced osteogenic differentiation but had no significant effect on hMSCs' proliferation. It probably exerted its functions through the signaling pathways of ERK1/2 as well as Wnt/*β*. Because DHA has low toxicity and costs, it might be regarded as an important drug for fracture treatment and tissue engineering.

## 1. Introduction

A great deal of fractures occurs every year due to trauma or pathology. Though a majority of fractures successfully heal in some months, approximately 5-10% of fractures show nonunion or delayed healing [1, 2], resulting in increased economic and psychological burdens on patients. There are several options to cure nonunion or delayed bone healing. Autologous bone grafting remains the gold standard, in which one or both iliac bones depending on the bone defect area are typically used. Although this method yields the highest recovery rate, it sometimes results in nonunion. Some orthopedists prefer allograft bone because it can reduce pain without autologous bone grafting. The observation of recombinant human bone morphogenetic protein-2 (BMP-2) is helpful for advancing the healing of fractures [3]. Nevertheless, BMP-2 also has limitations and defects. Thus, finding a novel, low-cost method with therapeutic efficiency to accelerate bone healing is urgently needed.

Recent findings have shown that certain cell types, such as human mesenchymal stem cells (hMSCs), can accelerate bone regeneration. MSCs possess multidirectional differentiation potential, including osteogenesis, adipogenesis, and chondrogenesis, making them hopeful seed cells to adopt within tissue engineering [4]. MSCs possess immunomodulatory and immunosuppressive properties and can modulate inflammation following injury through particular cytokines' secretion containing vascular endothelial development element, BMP-2, and the transformation of development element *β* (TGF-*β*) [5, 6]. The surrounding microenvironment activates the recruitment of MSCs for initial healing after a fracture, promotes the proliferation of MSCs, and finally induces differentiation into osteoblasts to facilitate bone healing [7].

Dihydroartemisinin (DHA), which is considered to be one of the active compounds within Artemisia annua, is classified into the family of sesquiterpene lactones. DHA shows important effectiveness against fibrosis, parasitic diseases, and malaria with minimal toxicity [8–10]. The functions of DHA have been intensely studied in recent years. According to some reports, antitumor activity is exerted by DHA in the cancers of humans, containing hepatomas, through the activation of caspase-independent and caspase-dependent cell death pathways [11–13]. In addition, Komaki and Zhou found that DHA suppressed estrogen deficiency-induced osteoporosis and osteoclast formation [14, 15]. Finally, Cao showed that DHA had a restrained impact on MSCs' hypertrophic and chondrogenic differentiation [16]. However, no research has reported the functions of DHA on hMSCs' osteogenic differentiation.

Recently, we characterized DHA's effect on hMSCs' proliferation as well as osteogenic differentiation and the underlying mechanism of action. Based on our findings and the low toxicity of DHA, it may have important applications in clinical settings.

## 2. Materials and Methods

### 2.1. Bone Marrow hMSC Extraction and Culture

hMSCs had been gained from three healthy donors' femoral bone marrow (24y, Female; 28y, male; 27y, male) at the Second Affiliated Hospital of Zhejiang University. The informed consent was given by the three donors before assembling their bone marrow. The experimental protocol adhered to the ethical standards of the Declaration of Helsinki and to global and domestic guidelines and was permitted by the institutional review board of the writer. hMSCs were cleaned from the femoral fracture fixation's intramuscular nailing, following a formerly depicted strategy [17]. The fraction of the mononuclear cell had been disconnected from the suspension via centrifugation over Ficoll-Paque at 2,500 rpm for half an hour at the time of gaining the femoral bone marrow suspension. The obtained cells were seeded in the growth medium of hMSC at room temperature including 5% CO2. The medium had been changed every three to four days, and the cells had been passed at about 80% confluence. hMSCs had been ready in the third passage for the tests which were reflected below.

### 2.2. Functions of DHA on hMSC Proliferation

hMSCs had been treated with 10 *μ*M, 1 *μ*M,100 nM, 10 nM or 0, and 1 nM DHA in the growth medium of hMSC for one day, two days, or three days and cultured within 96-well plates (10^3^/well). The Cell Counting Kit-8 (CCK-8) assay had been adopted for measuring the proliferation of cells which follow the instructions of the manufacturers. In brief, the medium had been substituted with 10 *μ*L of CCK-8 as well as 100 *μ*L of hMSC growth medium at the appointed time points. After incubation for approximately four hours at room temperature, a microplate reader had been adopted for measuring the absorbance at 450 nm.

Before the experiment, dimethyl sulfoxide (DMSO) was used to dissolve DHA immediately before use, and the final concentration of DMSO did not exceed 0.1%.

### 2.3. Osteogenic Differentiation Protocol and Alizarin Red Staining (ARS)

The cells had been seeded at a density of 3 × 10^4^/cm^2^ in 12-well culture plates and cultured with osteoinductive medium to induce osteogenic differentiation (OIM, the medium of modified Eagle of low-sugar Dulbecco, which was added by 50 mg ascorbic acid 2-phosphate/mL, 100 nM dexamethasone, and 10% fetal bovine serum). The cells had been kept by adding fresh osteogenic induction medium every two to three days.

ARS was used to evaluate mineral deposition. In brief, cells had been fixed for ten to twenty minutes under 4% paraformaldehyde at normal temperature, washed for three times by using the distilled water, treated for fifteen to thirty minutes by using ARS (0.5%) at normal temperature, and at last washed for three times by using distilled water. The stain was observed by incubating with 10% cetylpyridinium chloride (Sigma, Shanghai, China) for 1 h. Approximately 200 *μ*l of liquid was collected and transferred to 96-well plates. The absorbance was measured at 560 nm by using a microplate reader. The readings had been normalized to the overall concentrations of the proteins.

### 2.4. Western Blot Analysis

Cells had been separated by 10% sodium dodecyl sulfate polyacrylamide gel electrophoresis, lysed in RIPA buffer, and then transferred to a PVDF membrane. After being blocked for one to two hours at room temperature within 10% nonfat milk, the membranes had been incubated at 4°C overnight with antibodies particular to p-*β*-catenin (1:1,000, Cell Signaling Technology), p-ERK (1:1,000, Cell Signaling Technology), runt-related transcription factor 2 (RUNX2; 1:1,000, Cell Signaling Technology), and glyceraldehyde 3-phosphate dehydrogenase (GAPDH; 1:1,500, Cell Signaling Technology, Danvers, MA, America). After washing within Tris-buffered saline including Tween 20 (TBST) three times (10 minutes each), the membranes had been incubated by using horseradish-peroxidase-conjugated goat anti-rabbit IgG (1: 5,000, Cell Signaling Technology,) as a secondary antibody at normal temperature for an hour. After being washed with TBST for three times (ten minutes each), the immunoreactive bands had been noticed adopting a developed chemiluminescent detection reagent (Millipore) and further visualized through the explosion of the blot to X-ray film (Bio-Rad) for 0.2-2 minutes. The expression of proteins had been quantified through the determination of the ratio of the proteins' absorbance to that of the inner GAPDH control.

### 2.5. RNA Isolation and qPCR

hMSCs were cultured by using 1 *μ*M DHA as well as OIM for three to seven days after being implanted into 6-well plates (4 × 10^4^ cells/well). First, RNAiso reagent (Takara, Dalian, China) was used to extract the total cellular RNA, which was quantified through the measurement of the absorbance at 260 nm. Second, reverse transcription had been implemented by adopting PrimeScript RT Master Mix (Takara) in accordance with the instructions of the manufacturer. Every gene transcript had been quantified by qPCR adopting Power SYBR Green PCR Master Mix (Takara) on an ABI StepOnePlus System. Next, the target genes' mRNAs were quantified according to the following conditions: 95°C for half a minute followed by 45 cycles of 60°C for half a minute and 95°C for 5 seconds. The primer sequences adopted have been presented within Table 1. GAPDH or 18S had been adopted as an inner control for adjusting for the different points among the samples. The concentrations had been measured by adopting the 2–ΔΔCt strategy. Every primer adopted within the test had been synthesized by Sangon Biotech.

### 2.6. Immunofluorescence Analysis

The cells had been cultured with OIM and 1 *μ*M DHA for 72 hours before being seeded within a 12-well plate. Fluorescence microscopy (Leica, Wetzlar, Germany) was used to measure RUNX2, collagen *α*1 type I (COL1A1), p-ERK, and p-*β*-catenin. Briefly, cells had been settled at normal temperature within 4% paraformaldehyde for at least 15 min, washed with distilled water three times, blocked in 5% bovine serum albumin as well as 0.03% Triton X-100 for half an hour, washed with distilled water three times, and incubated overnight with anti-p-*β*-catenin (1:1500, Cell Signaling Technology), anti-p-ERK (1:500, Cell Signaling Technology), anti-COL1A1 (1:500, Abcam, Cambridge, UK), or anti-RUNX2 (1:1,600, Cell Signaling Technology). Cells had been incubated subsequently with a fluorescence-conjugated secondary antibody (Beyotime) for an hour. Cell nuclei had been stained with 4′, 6-diamidino-2-phenylindole for 5 minutes. Cell immunofluorescence was noticed adopting fluorescence microscopy (Leica).

### 2.7. Statistical Analysis

Statistical analysis had been implemented adopting SPSS statistical software for Windows (ver. 17.0, SPSS Inc., Chicago, IL, USA). Statistical significance had been decided adopting the Wilcoxon nonparametric experiments as well as Mann–Whitney. The resources have been shown as the means ± standard deviation. A value of p ≤ 0.05 had been regarded to imply statistical significance.

## 3. Results

### 3.1. DHA, at Low Concentrations, Had No Significant Effect on Cell Proliferation

**Figure 1:**
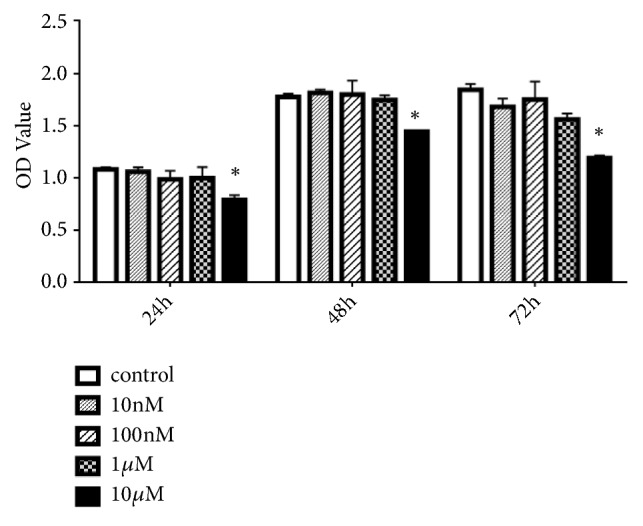
Effects of different concentrations of dihydroartemisinin (DHA) on human mesenchymal stem cell (hMSC) expansion. *∗*P (10 *μ*M) < 0.05 vs. the control group.

For the purpose of assessing DHA's function on hMSCs' viability and proliferation, we used CCK-8 assay (Dojindo Laboratories). Basal culture medium had been adopted for treating hMSCs for a day, two days, or three days with different concentrations of DHA (ranging from 1 nM to 10 *μ*M). DHA treatment at 1 nM to 1 *μ*M did not affect greatly the proliferation of hMSCs (P > 0.05, Figure 1) but showed a prohibitive impact at 10 *μ*M (P < 0.05, Figure 1).

### 3.2. Function of DHA on the Osteogenic Differentiation of hMSCs

**Figure 2:**
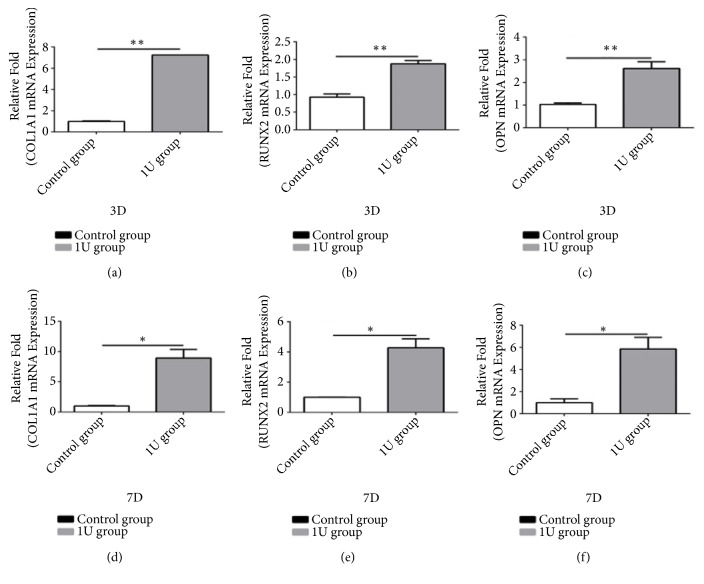
Gene expression levels of collagen *α*1 type I (COL1A1), osteopontin (OPN), and runt-related transcription factor 2 (RUNX2) in the control and dihydroartemisinin (DHA, 1 *μ*M) groups on days 3 and 7(a–f). *∗*P < 0.05 vs. the control group.

We used qPCR for evaluating DHA's role in hMSCs' osteogenic differentiation. Osteospecific genes' greatly higher expression degrees exist, containing osteopontin, COL1A1, and RUNX2, in the 1 *μ*M DHA group compared with that within the control group on the third and seventh days (P < 0.05, Figure 2).

Calcium deposition was tested by ARS, and the stained regions had been quantified through calculating the absorbance at 560 nm. It was found that there was an increasing number of calcium deposition in the OIM of the 1 *μ*M DHA group instead of the control group at 14 days.

### 3.3. DHA Increased the Phosphorylation of *β*-Catenin in hMSCs

**Figure 3:**
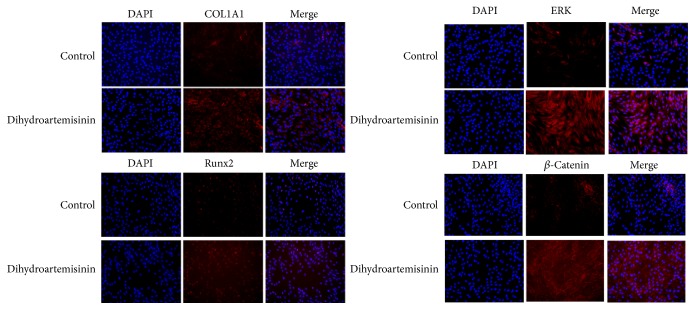
Immunofluorescence of runt-related transcription factor 2 (RUNX2), collagen *α*1 type I (COL1A1), phosphorylated (p)-ERK, and p-*β*-catenin after 72 h culture in osteoinductive medium (OIM) in human mesenchymal stem cells (hMSCs) with or without dihydroartemisinin (DHA, 1 *μ*M).

DHA's significant impact was explored in the signaling pathway of *β*-catenin during osteogenesis using immunofluorescence, Western blot, and ARS analyses. The p-*β*-catenin degrees had been much lower in the control group than in the DHA-treated group after 24 h in osteoculture (Figure 3).

**Figure 4:**
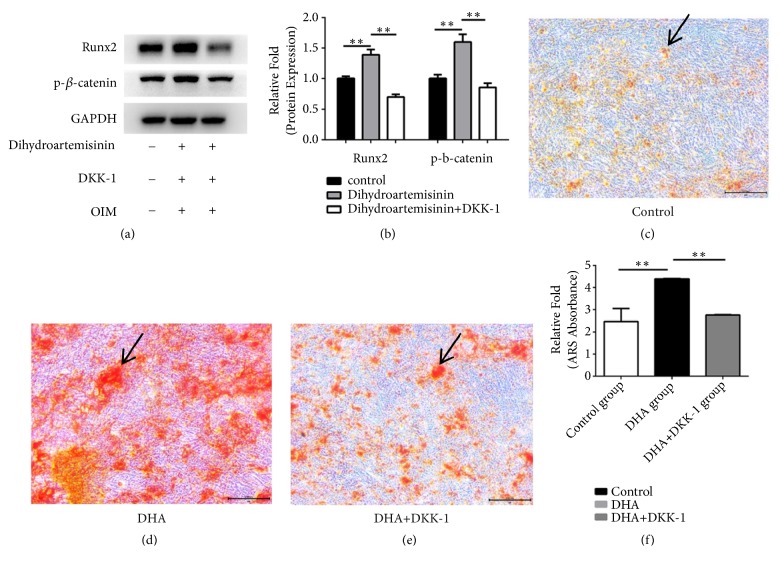
(a) Human mesenchymal stem cells (hMSCs) were cultured in osteoinductive medium (OIM), OIM + 1 *μ*M dihydroartemisinin (DHA), or OIM + 1 *μ*M DHA + DKK-1, after which the expression levels of p-*β*-catenin and runt-related transcription factor 2 (RUNX2) were measured using Western blotting. (b) Protein expression levels were normalized to glyceraldehyde 3-phosphate dehydrogenase (GAPDH). *∗*P < 0.05 vs. the control OIM group(c–e). hMSCs were cultured in OIM, OIM + 1 *μ*M DHA, or OIM +1 *μ*M DHA + DKK-1 and stained with alizarin red on day 14. (f) Mineralization was quantified by the extraction of alizarin red-stained cells. *∗*P < 0.05 vs. the OIM and OIM + DHA + DKK-1 groups. Black arrows represent calcium deposits.

Western blot analysis revealed that DHA, increased p-*β*-catenin expression, and DKK-1 combined with 1 *μ*M DHA inhibited the increase in RUNX2 protein expression (P < 0.05, Figures 4(a) and 4(b)).

Moreover, cells treated with DKK-1 (for inhibiting the signaling pathway of Wnt/*β*-catenin) exhibited less calcium deposition at day 14 after osteogenic differentiation (P < 0.05, Figures 4(c)–4(f)).

### 3.4. DHA Increased the Phosphorylation of Phosphorylated (p)-ERK in hMSCs

There is no doubt that the signaling pathway of ERK is very significant in promoting the osteogenesis of hMSC. Therefore, DHA's probable impact was evaluated on the signaling pathway of ERK by adopting immunofluorescence.

After 72 h in osteogenic induction medium, we observed a significant increase in the protein expression degrees in COL1A1 as well as RUNX2 within the DHA-treated group by comparing with the control group. In addition, the p-ERK content had been much higher within the DHA-treated hMSC group after 24 h in osteoculture (Figure 3).

**Figure 5:**
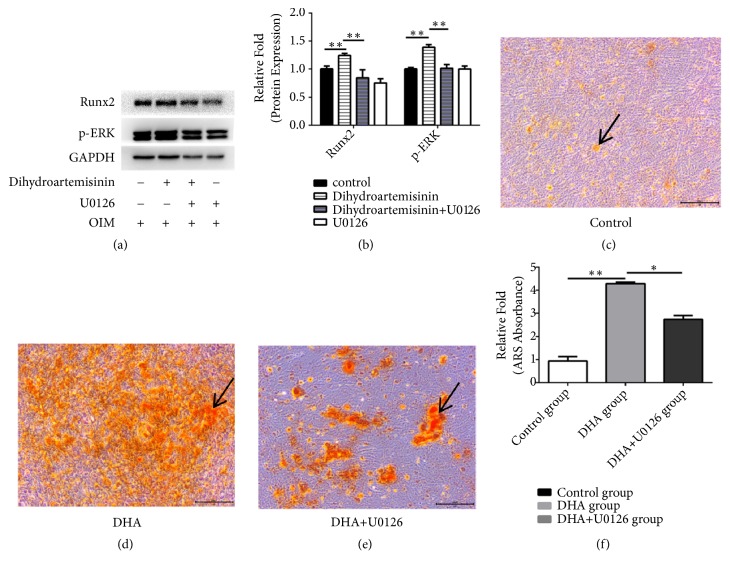
(a) Human mesenchymal stem cells (hMSCs) were cultured in osteoinductive medium (OIM), OIM + 1 *μ*M dihydroartemisinin (DHA), OIM + 1 *μ*M DHA + U0126, or OIM + U0126. The levels of phosphorylated (p)-ERK and runt-related transcription factor 2 (RUNX2) were measured using Western blotting. (b) Protein expression levels were normalized to glyceraldehyde 3-phosphate dehydrogenase (GAPDH). *∗*P < 0.05 vs. the control OIM group(c–e). hMSCs were cultured in OIM, OIM + 1 *μ*M DHA, or OIM + 1 *μ*M DHA + U0126 and stained with alizarin red on day 14. (f) Mineralization was quantified by the extraction of alizarin red-stained cells. *∗*P < 0.05 vs. OIM and OIM + DHA+ U0126 groups. Black arrows represent calcium deposits.

Western blot analysis had been adopted for examining cells cultured with OIM, OIM + 1 *μ*M DHA, OIM + 1 *μ*M DHA and U0126 (5 ng/ml, an ERK inhibitor), or U0126 alone for 3 days. An important increase was found in the expression of p-ERK as well as RUNX2 in the 1 *μ*M DHA group, and treatment with U0126 alone inhibited RUNX2 and p-ERK expression (P < 0.05, Figures 5(a) and 5(b)).

Finally, cells treated with U0126 (to inhibit the ERK signaling pathway) exhibited less calcium deposition at day 14 after osteogenic differentiation (P < 0.05, Figures 5(c)–5(f)).

## 4. Discussion

DHA was recently shown to have protective impacts on the nervous, digestive, and respiratory systems [18, 19]. In 2017, Cao [16] reported that DHA exerted inhibitory impacts on MSCs' hypertrophic and chondrogenic differentiation. A recent study also noted that DHA efficiently prevented breast cancer-induced osteolysis and prohibited osteoclastogenesis [20]. Considering these observations, we hypothesized that DHA influences the osteogenic differentiation of MSCs.

It was illustrated in the paper that though DHA did not influence significantly the proliferation of hMSCs at low concentrations, it advanced osteogenic differentiation via two signaling pathways. According to what we have known, it is the primary research for illustrating that DHA induces osteogenic differentiation. Although there has been no report indicating the most effective concentration for osteogenic differentiation, we drew insight from previous studies. It is crucial to note that proliferation was inhibited when treated with high concentrations of DHA (100 *μ*M) [16]. Furthermore, Zhou [14] showed that DHA prohibited greatly the formation of osteoclast at doses over 0.5 *μ*M, with no cytotoxic effects on bone marrow-derived macrophages. Hence, the effects of DHA on hMSCs may be dose dependent. When considering the inhibition of proliferation at high concentrations of DHA and the acceleration of osteogenic differentiation, 1 *μ*M DHA was selected for the later tests of osteogenic differentiation (P < 0.05, Figures 1 and 2).

There is a relationship between MSC osteogenic differentiation and numerous signaling pathways, containing the phosphatidylinositide-3 kinase (PI3K)/Akt, Wnt/*β*, ERK1/2, and BMP-Smad pathways [21–24]. In this research, it was illustrated that the signaling pathways of Wnt/*β* and ERK1/2 had some relationships with DHA's capacity for inducing MSC osteogenic differentiation. The pathway of ERK is involved in the protein kinase pathway which is activated by mitogen. The adipose-derived stem cells' osteogenic reaction would be decreased by ERK1/2 signaling pathway inhibition [25]. Nevertheless, Lund et al. [26] pointed that the prohibition of the signaling pathway of ERK1/2 augmented the osteogenic reaction in hMSCs, which may be dependent on the cell type or treatment used. In our research, DHA could promote expression of RUNX2 and the ERK. RUNX2 is a key transcription factor for osteogenesis. However, when using U0126 to inhibit the ERK1/2 signaling pathway, we found that the matrix mineralization and protein expression of RUNX2 were adequately decreased, demonstrating that the pathway of ERK1/2 significantly affects DHA-induced osteogenic differentiation.

Another significant pathway that is included in osteogenesis is the Wnt/*β*-catenin pathway. Being a member of a family of secreted proteins, Wnt may activate *β*-catenin at a downstream location and bind to seven-pass transmembrane Frizzled receptors [27]. The signaling pathway of Wnt/*β*-catenin is considered to be rather significant in cell proliferation, differentiation, growth, regeneration, self-renewal, and the determination of cell fate [28]. Krishnan found that canonical Wnt signaling via *β*-catenin had been induced essentially during fracture healing and has thus been considered to be osteoinductive. The signaling pathway of Wnt is included in promoting the impacts of osteoblast as well as osteoblastogenesis, leading to improvements in adult bone mass [29]. In our study, DHA induced *β*-catenin and RUNX2 expression. Using DKK-1 to control *β*-catenin activation, calcium deposition and the expression of RUNX2 were slightly inhibited. Therefore, the pathway of Wnt/*β*-Catenin has been partly involved in the hMSCs' osteogenic differentiation induced by DHA.

There are some limitations about the research. First, we assessed hMSCs' potential mechanisms as well as their osteogenic differentiation just by using a concentration of 1 *μ*M DHA. An increasing number of researches have been warranted for illustrate the impacts of diversified concentrations of DHA. Second, we indicated that both the signaling pathways of Wnt/*β*-catenin and ERK had been involved in the DHA-induced osteogenic differentiation of hMSCs, but it is still not clear if there is crosstalk between Wnt/*β* and ERK signaling pathways existing. Third, we did not conduct an in vivo experiment to prove that DHA promoted fracture healing.

**Figure 6:**
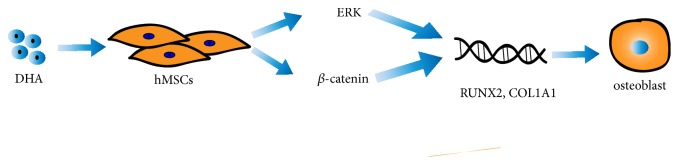
Schematic diagram of the signaling pathways involved in osteoblast differentiation induced by DHA. DHA promotes osteoblast differentiation through the ERK and Wnt/*β*-catenin signaling pathways.

In conclusion, our study indicates that DHA has no significant effect on the proliferation of hMSCs but enhances osteogenic differentiation via the signaling pathways of Wnt/*β* as well as ERK1/2 (Figure 6).

## Figures and Tables

**Table 1:** Sequences of primers used for quantitative real-time PCR.

Gene	Forward primer	Reverse primer
RUNX2	CAAGTGGCCAGGTTCAACGA	TGTGAAGACCGTTATGGTCAAAGG
COL1A1	CCTGCTGGCAAGAGTGGTGAT	CAAGTTCCGGTGTGACTCGTG
OCN	AGGACCCTCTCTCTGCTCAC	GCTCACACACCTCCCT
GAPDH	AATGGGCAGCCGTTAGGAAA	GCGCCCAATACGACCAAATC
18S	GACTCAACACGGGAAACCTCAC	CCAGACAAATCGCTCCACCAAC

## Data Availability

The article data used to support the findings of this study are available from the corresponding author upon request.

## References

[1] Claes L., Recknagel S., Ignatius A. (2012). Fracture healing under healthy and inflammatory conditions. *Nature Reviews Rheumatology*.

[2] Gómez-Barrena E., Rosset P., Lozano D., Stanovici J., Ermthaller C., Gerbhard F. (2015). Bone fracture healing: cell therapy in delayed unions and nonunions. *Bone*.

[3] Govender S., Csimma C., Genant H. K. (2002). Recombinant human bone morphogenetic protein-2 for treatment of open tibial fractures: a prospective, controlled, randomized study of four hundred and fifty patients. *The Journal of Bone & Joint Surgery—American Volume*.

[4] Klimczak A., Kozlowska U. (2016). Mesenchymal stromal cells and tissue-specific progenitor cells: Their role in tissue homeostasis. *Stem Cells International*.

[5] Marcucio R. S., Nauth A., Giannoudis P. V. (2015). Stem cell therapies in orthopaedic trauma. *Journal of Orthopaedic Trauma*.

[6] Henrich D., Seebach C., Nau C. (2016). Establishment and characterization of the Masquelet induced membrane technique in a rat femur critical-sized defect model. *Journal of Tissue Engineering and Regenerative Medicine*.

[7] Gibon E., Lu L., Goodman S. B. (2016). Aging, inflammation, stem cells, and bone healing. *Stem Cell Research & Therapy*.

[8] Hanboonkunupakarn B., White N. J. (2016). The threat of artemisinin resistant malaria in Southeast Asia. *Travel Medicine and Infectious Disease*.

[9] Kakuru A., Jagannathan P., Muhindo M. K. (2016). Dihydroartemisinin–piperaquine for the prevention of malaria in pregnancy. *The New England Journal of Medicine*.

[10] Nji A. M., Ali I. M., Moyeh M. N. (2015). Randomized non-inferiority and safety trial of dihydroartemisin-piperaquine and artesunateamodiaquine versus artemether-lumefantrine in the treatment of uncomplicated Plasmodium falciparum malaria in Cameroonian children. *Malaria Journal*.

[11] Feng X., Li L., Jiang H., Jiang K., Jin Y., Zheng J. (2014). Dihydroartemisinin potentiates the anticancer effect of cisplatin via mTOR inhibition in cisplatin-resistant ovarian cancer cells: involvement of apoptosis and autophagy. *Biochemical and Biophysical Research Communications*.

[12] Zhang Z. S., Wang J., Shen Y. B. (2015). Dihydroartemisinin increases temozolomide efficacy in glioma cells by inducing autophagy. *Oncology Letters*.

[13] Qu C., Ma J., Liu X. (2017). Dihydroartemisinin exerts anti-tumor activity by inducing mitochondrion and endoplasmic reticulum apoptosis and autophagic cell death in human glioblastoma cells. *Frontiers in Cellular Neuroscience*.

[14] Zhou L., Liu Q., Yang M. (2016). Dihydroartemisinin, an anti-malaria drug, suppresses estrogen deficiency-induced osteoporosis, osteoclast formation, and rankl-induced signaling pathways. *Journal of Bone and Mineral Research*.

[15] Komaki S., Sakai E., Fukuma Y., Nishishita K., Okamoto K., Tsukuba T. (2018). Dihydroartemisinin represses osteoclastogenesis of bone marrow macrophages through reduced NFATc1 expression and impaired phosphorylation of I*κ*B*α*. *Biomedical Research*.

[16] Cao Z., Liu C., Bai Y. (2017). Inhibitory effect of dihydroartemisinin on chondrogenic and hypertrophic differentiation of mesenchymal stem cells. *American Journal of Translational Research*.

[17] Zong C., Xue D., Yuan W. (2010). Reconstruction of rat calvarial defects with human mesenchymal stem cells and osteoblast-like cells in poly-lactic-co-glycolic acid scaffolds. *European Cells and Materials*.

[18] Wei M., Xie X., Chu X., Yang X., Guan M., Wang D. (2013). Dihydroartemisinin suppresses ovalbumin-induced airway inflammation in a mouse allergic asthma model. *Immunopharmacology and Immunotoxicology*.

[19] Sun H., Meng X., Han J. (2013). Anti-cancer activity of DHA on gastric cancer—an in vitro and in vivo study. *Tumor Biology*.

[20] Feng M., Hong J., Wang Q. (2016). Dihydroartemisinin prevents breast cancer-induced osteolysis via inhibiting both breast caner cells and osteoclasts. *Scientific Reports*.

[21] Simann M., Le Blanc S., Schneider V. (2017). Canonical FGFs prevent osteogenic lineage commitment and differentiation of human bone marrow stromal cells Via ERK1/2 signaling. *Journal of Cellular Biochemistry*.

[22] Baker N., Sohn J., Tuan R. S. (2015). Promotion of human mesenchymal stem cell osteogenesis by PI3-kinase/Akt signaling, and the influence of caveolin-1/cholesterol homeostasis. *Stem Cell Research & Therapy*.

[23] Tornero-Esteban P., Peralta-Sastre A., Herranz E. (2015). Altered expression of wnt signaling pathway components in osteogenesis of mesenchymal stem cells in osteoarthritis patients. *PLoS ONE*.

[24] Li A., Xia X., Yeh J. (2014). PDGF-AA promotes osteogenic differentiation and migration of mesenchymal stem cell by down-regulating PDGFR*α* and derepressing BMP-Smad1/5/8 signaling. *PLoS ONE*.

[25] Liu Q., Cen L., Zhou H. (2009). The role of the extracellular signal-related kinase signaling pathway in osteogenic differentiation of human adipose-derived stem cells and in adipogenic transition initiated by dexamethasone. *Tissue Engineering Part A*.

[26] Lund A. W., Stegemann J. P., Plopper G. E. (2009). Inhibition of ERK promotes collagen gel compaction and fibrillogenesis to amplify the osteogenesis of human mesenchymal stem cells in three-dimensional collagen i culture. *Stem Cells and Development*.

[27] Gruber J., Yee Z., Tolwinski N. S. (2016). Developmental drift and the role of wnt signaling in aging. *Cancers*.

[28] Shi J., Chi S., Xue J., Yang J., Li F., Liu X. (2016). Emerging role and therapeutic implication of wnt signaling pathways in autoimmune diseases. *Journal of Immunology Research*.

[29] Krishnan V., Bryant H. U., MacDougald O. A. (2006). Regulation of bone mass by Wnt signaling. *The Journal of Clinical Investigation*.

